# The Independent Biological Activity of *Bacillus thuringiensis* Cry23Aa Protein Against *Cylas puncticollis*

**DOI:** 10.3389/fmicb.2020.01734

**Published:** 2020-07-22

**Authors:** Patricia Hernández-Martínez, Ayda Khorramnejad, Katterine Prentice, Ascensión Andrés-Garrido, Natalia Mara Vera-Velasco, Guy Smagghe, Baltasar Escriche

**Affiliations:** ^1^Laboratory of Biotechnological Control of Pest, ERI de Biotecnología y Biomedicina, Department of Genetics, Universitat de València, Burjassot, Spain; ^2^Laboratory of Biological Control of Pest, Department of Plant Protection, College of Agriculture and Natural Resources, University of Tehran, Karaj, Iran; ^3^Laboratory of Agrozoology, Department of Plants and Crops, Faculty of Bioscience Engineering, Ghent University, Ghent, Belgium; ^4^Laboratory Membrane Proteins, ERI de Biotecnología y Biomedicina, Department of Biochemistry and Molecular Biology, Universitat de València, Burjassot, Spain

**Keywords:** binary toxin, Cry37Aa, sweet potato weevils, mode of action, insecticidal proteins, bioassay, binding assay

## Abstract

The Cry23Aa/Cry37Aa proteins from *Bacillus thuringiensis* (Bt) have been described toxic to *Cylas puncticollis* larvae. In general, it is believed that Cry23Aa and Cry37Aa act jointly to exert the insecticidal activity, while there is no evidence of their toxicity individually. Therefore, in the present study, the contribution of each protein in the insecticidal activity toward *C. puncticollis* larvae has been assessed. The results showed that both proteins were toxic for *C. puncticollis* larvae when tested individually. Contrary to what was claimed previously, our results suggest that the presence of both proteins is not necessary to exert toxicity against *C. puncticollis* larvae. Also, the binding behavior of Cry23Aa protein to midgut receptors of *C. puncticollis* larvae has been determined. According to our results, Cry23Aa binds to *C. puncticollis* brush border membrane vesicles (BBMV) specifically and independently of Cry37Aa. Due to the lack of common binding sites, Cry23Aa can be pyramided with Cry3Aa protein for better management of *C. puncticollis*.

## Introduction

Sweet potato, *Ipomoea batatas* L., is one of the mostly grown crops in Africa. Several serious biotic and abiotic factors threaten the sweet potato production. Sweet potato weevil, *Cylas puncticollis* Boheman (Coleoptera: Brentidae), is one of the most economically important sweet potato insect pests in Africa (Chalfant et al., 1990). The high infestation percent of sweet potato to *Cylas* spp. resulted in a dramatic reduction of the tuber yield and a high percentage of unmarketable crops (Sorensen, 2009). Tremendous economic losses occur when *Cylas* spp. larvae tunnel inside the sweet potatoes, subsequently the occurrence of microbial contamination and terpenoid odor production (Smit et al., 1997). Due to the importance of this tuber crop in human consumption, livestock feed, and industrial production (Hazra et al., 2011), finding an appropriate strategy in controlling *C. puncticollis* is highly demanded.

The control of *C. puncticollis* is difficult, due to the special behavior of this insect pest. The egg-laying, larval development, and feeding *Cylas* larvae occur inside the sweet potatoes, which is unavailable for insecticides dispersed on the surface of sweet potatoes. Many strategies have been developed for the management of this pest including; cultural methods such as clean planting vines, crop rotation, early planting, and harvesting (Ochieng et al., 2017), mass trapping using sex pheromones (Smit et al., 1997; Downham et al., 2001; Reddy et al., 2014a), breeding the resistance cultivars of *I. batatas* (Stevenson et al., 2009; Anyanga et al., 2013; Ochieng et al., 2017), fumigants and chemical control (Hwang, 2000), and phytosanitary treatment by X-ray irradiation (Follett, 2006). Amongst all the management approaches, biological control focusing on soil inhabitant insect pathogens; fungi, bacteria, and nematode have better effectiveness in controlling *Cylas* spp. (Ondiaka et al., 2008). Entomopathogenic fungi *Beauveria bassiana* and *Metarhizium anisopliae* (Alcázar et al., 1997; Reddy et al., 2014b) and entomopathogenic nematodes *Steinernema carpocapsae* and *Heterorhabditis bacteriophora* (Jansson and Lecrone, 1997) have been used for controlling *Cylas* spp. Moreover, the efficiency of different *Bacillus thuringiensis* insecticidal proteins in controlling *Cylas* spp. has been previously shown (Morán et al., 1998; Ekobu et al., 2010; Rukarwa et al., 2014).

The entomopathogenic bacterium *B. thuringiensis* (Bt) produces diverse groups of insecticidal proteins toxic to many insects from different orders (Palma et al., 2014). The well-known crystal proteins (Cry) are produced during the sporulation at the stationary phase of growth (de Maagd et al., 2003). It is generally accepted that the mode of action of these proteins starts after the ingestion of the protein by the insect. Then, these proteins are solubilized, activated by the action of digestive enzymes, bind to specific receptors in the brush border of epithelial midgut cells, and form pores in the membrane which eventually lead to septicemia and insect death (Palma et al., 2014).

Nowadays, some studies have been performed to identify toxic Cry proteins to *Cylas* spp. larvae (Ekobu et al., 2010) such as the coleopteran-active Cry proteins; Cry3Aa3, Cry3Bb3, Cry3Ca1, Cry7Aa1, and Cry23Aa/Cry37Aa (Ekobu et al., 2010; Rukarwa et al., 2014). The development and efficiency of Bt transgenic sweet potato carrying a single Bt *cry* gene have been evaluated (Morán et al., 1998; Rukarwa et al., 2014). Whereas, none of the transgenic sweet potatoes controlled *Cylas* spp. efficiently due to low *cry* gene expression in Bt crop. Alternatively, a combination of several *cry* genes in the same Bt crop would serve to delay the onset of resistance when more than one insecticidal protein is active against the same insect species. This rationale is based on the occurrence of different binding sites for the proteins that are pyramided, since if the pyramided proteins share a binding site in the midgut of the larva, a single mutation could confer cross-resistance to these proteins. By the aim of employing more than one Bt toxin, competition binding experiments have been previously performed by other authors in the midgut of *C. puncticollis* larvae and have provided models for the binding sites to predict or to explain patterns of cross-resistance or multiple resistance (Hernández-Martínez et al., 2014, 2016). As a result, the occurrence of shared binding sites for Cry3Ca, Cry3Ba, and Cry7Aa proteins to *C. puncticollis* brush border membrane vesicles (BBMV) has been described (Hernández-Martínez et al., 2014). In addition, Cry3Aa shared at least one common binding site with Cry3Ca on *C. puncticollis* BBMV (Hernández-Martínez et al., 2016). Hence, co-expression of two or more non-homologous *cry* genes as a gene-pyramiding strategy targeting *C. puncticollis* might offer promising improvement in sweet potato weevil management. But, before the expression of multiple Bt *cry* genes in sweet potato, the insecticidal activity and different mechanisms of action of Cry proteins must be evidenced.

The Cry23Aa/Cry37Aa proteins were characterized as binary toxin based on the fact that both genes were located in the same operon. Moreover, the toxicity against different coleopterans was achieved by expressing both genes in a recombinant *B. thuringiensis* strain (Donovan et al., 2001). Later the insecticidal activity of these proteins was assessed against different coleopteran pests (Donovan et al., 2001; Weathersbee et al., 2006; Contreras et al., 2013; Gindin et al., 2014). Even though the crystal structure of both proteins mainly consists of β-strands, they belong to different homology groups. Crystal structure of Cry23Aa resembles that of Mtx-type toxins, Cry51, Cry35, PS2Aa1, and aerolysin-type β-pore-forming-toxins (de Maagd et al., 2003; Akiba and Okumura, 2017). Whereas Cry37Aa shows structural homology with Cry34Ab1 (Rydel et al., 2001; de Maagd et al., 2003; Berry and Crickmore, 2017) and belongs to the “rich in beta sheets toxins” class (Berry and Crickmore, 2017). However, there is no information about the mode of action of these proteins. In the present work, the Cry23Aa/Cry37Aa protein produced in *B. thuringiensis* strain was characterized in more detail in terms of insecticidal activity, proteolytic processing, the interaction between components in solution based on the size exclusion chromatography and binding behavior. Moreover, to determine whether both proteins are required for the toxic effect, the contribution of Cry23Aa and Cry37Aa proteins, individually produced in *Escherichia coli* cells, in the insecticidal activity of the binary proteins was assessed against *C. puncticollis* larvae. Eventually, the binding behavior of Cry23Aa alone or in the combination with Cry37Aa was studied. Finally, by the aim of the combination of two or more *cry* genes in Bt sweet potato, the binding properties of Cry23Aa (alone and in combination with Cry37Aa) and Cry3Aa proteins have been studied by performing binding competition experiments.

## Materials and Methods

### Insects

A colony of the African sweet potato weevil *C. puncticollis* was reared in plastic cages and placed into climate-controlled incubators at 27°C, 65% RH under a 16:8 light:dark regimen. The weevils were kept for feeding and oviposition on sweet potato storage roots and changed every week for fresh roots.

### Cloning of Cry23Aa and Cry37Aa in *E. coli*

The *B. thuringiensis cry23Aa* and *cry37Aa* genes (NCBI accession No.AF038048 and AF038049, respectively) were codon-optimized and synthesized by GenScript (NJ, United States). The sequences were modified replacing rare codons for the most abundant codons of *E. coli*. Each gene was supplied as a lyophilized double-stranded DNA in a pET-15b vector between *Xho*I and *Bam*HI cut sites, resulting in a fusion protein with a 6-histidine tag at the N-terminus. The sequence of both codon-optimized genes was confirmed by Sanger sequencing using plasmid universal primers of the T7 promoter and T7 terminator at the Genomics Facility of SCSIE at the University of Valencia.

### Cry Proteins Preparation

Cry proteins used in the present work were obtained from different sources. Cry3Aa and Cry23Aa/Cry37Aa proteins were obtained from the *B. thuringiensis* strain 4AA1 provided by the Bacillus Genetic Stock Center (BGSC), United States and EG10327 (Ref. No. NRRL B-21365) obtained from the Agricultural Research Culture Collection, Northern Regional Research Laboratory (NRRL), United States, respectively. Both strains were grown in CCY medium (Stewart et al., 1981) for 48 h at 29°C in constant agitation. Spores and crystals were collected by centrifugation (10 min, 16,000 × *g* at 4°C) and washed three times with 1 M NaCl, 10 mM ethylenediaminetetraacetic acid (EDTA) and twice with 10 mM KCl. Crystal proteins were solubilized in 50 mM carbonate buffer (Na_2_CO_3_, pH 12), 10 mM dithiothreitol (DTT) for 16 h at 37°C.

Cry23Aa and Cry37Aa proteins were produced in recombinant *E. coli* BL21 (DE3). Recombinant *E. coli* cells were grown in 10 ml of Luria-Bertani (LB) broth supplemented with ampicillin (100 μg/ml) and incubated with shaking at 37°C for 3 h until the optical density of 600 nm reached 0.6. Cultures were induced with 0.4 mM isopropyl-β-D-thiogalactopyranoside (IPTG) and let it grow for 16 h at 37°C. Cells were recovered by centrifugation at 12,000 × *g* for 20 min at 4°C. The presence of both proteins in the cultured was determined by sodium dodecyl sulfate 15% polyacrylamide gel electrophoresis (SDS-PAGE) as described by Laemmli (1970). Cry1Ab protein was obtained from a recombinant *E. coli* strain kindly supplied by R. A. de Maagd (Wageningen Plant Research, Wageningen University) and produced as described elsewhere (Sayyed et al., 2005).

Inclusion bodies containing Cry proteins were purified and solubilized as follows. After cell lysis, the pellets were recovered by centrifugation at 12,000 × *g* for 20 min and then washed five times with washing buffer (20 mM Tris–HCl, pH 8.5 mM EDTA, 100 mM NaCl). Protoxin solubilization was performed by incubation of inclusion bodies at 37°C in solubilization buffer (50 mM sodium carbonate, pH 12) containing 10 mM DTT. After 3 h, the solubilized protoxin was separated from insoluble debris by centrifugation at 12,000 × *g* for 20 min. As a control, the empty *E. coli* BL21 (DE3) strain was cultured and processed in the same way as described above for the Cry23Aa or Cry37Aa producing strains. Proteins were kept at −20°C until used.

### Cry Protein Activation

The activation of Cry23Aa/Cry37Aa protoxins from *B. thuringiensis* was carried out by incubating the proteins with trypsin (type I) (Sigma-Aldrich) or with *C. puncticollis* midgut juice proteases. For midgut juice activation, Cry23Aa/Cry37Aa (6 μg) were incubated with gut fluid from *C. puncticollis* with a ratio of 1:1 (wt protease/wt protoxin) in a final volume of 40 μl in phosphate buffered saline (PBS), for 30, 60, and 180 min at 30°C. Trypsin activation was performed by incubation of Cry23Aa/Cry37Aa (ratio 1:10) at 37°C using the same time intervals. At the end of the activation process, the processed samples were snap frozen in liquid nitrogen and subjected to 15% SDS-PAGE.

Cry3Aa protoxin was activated with bovine pancreas trypsin (1:5 wt/wt) for 3 h at 37°C. The concentration of Cry3Aa, Cry23Aa, and Cry37Aa active proteins was estimated by densitometry using TotalLab Quant program version 12.3, employing bovine serum albumin (BSA), as a standard protein. The activated proteins were kept at −20°C until used.

### Size Exclusion Chromatography

Gel filtration chromatography was carried out with an ÄKTA explorer 100 chromatography system (GE Healthcare) with a Superdex 75 10/300 GL column (GE Healthcare Life Sciences, Uppsala, Sweden) equilibrated and eluted with 50 mM sodium carbonate buffer, pH 12, at a flow rate of 0.5 ml/min. To estimate the apparent molecular weight of the chromatographic peaks, the column was calibrated with a protein molecular weight standards kit (HMW calibration kit, GE Healthcare Life Sciences). Then, the samples consisted of Cry23Aa/Cry37Aa activated with trypsin were injected into the column.

### Anion-Exchange Purification

For binding assays, the trypsin-activated Cry23Aa/Cry37Aa from *B. thuringiensis* was dialyzed in 20 mM Tris–HCl, pH 8.6, and filtered prior to anion-exchange purification in a HiTrap Q HP column using an ÄKTA explorer 100 chromatography system (GE Healthcare, United Kingdom). Proteins were eluted by applying a linear gradient (from 0.1 to 0.7) of 1 M NaCl. The gradient length was 20 bed columns (100 ml). The eluted fractions from the column were individually analyzed by SDS-PAGE. The fractions corresponding to the trypsin-activated proteins were pooled and kept at −20°C until used.

### Protein Fingerprinting

To confirm the identity of the recombinant proteins based on their peptide profiles, a protein mass fingerprinting was performed. Solubilized Cry23Aa and Cry37Aa from recombinant *E. coli* strains were run in an SDS-PAGE and the bands corresponding approximately to molecular masses of 30 and 14 kDa were excised. Moreover, as a control, the bands corresponding to the Cry23Aa and Cry37Aa proteins produced in *B. thuringiensis* strain EG10327 were also analyzed. Samples were digested with trypsin and analyzed by LC-MS/MS at the Proteomics Core Facility of SCSIE, University of Valencia, with a mass spectrometer in a nanoESI Q-TOF (TripleTOFTM 5600, AB SCIEX). The fingerprinting data were used to search for protein candidates using MASCOT software.

### Toxicity of Cry Proteins Against Sweet Potato Weevils

Bioassays using separately the recombinant proteins produced in *E. coli* cells or the Cry23Aa/Cry37Aa proteins produced by the *B. thuringiensis* strain EG10327 were performed to test whether the requirement of both proteins is needed for the insecticidal activity against *C. puncticollis*. Second instar larvae were gently removed from storage sweet potato roots after 9 days of oviposition for oral feeding experiments. The artificial diet was prepared according to the protocol by Ekobu et al. (2010) with modified agar concentration to 60 g/L diet. Proteins were mixed with the diet at a concentration of 1 and 5 μg/g diet and poured into Petri dishes to cool down at room temperature. Fifteen larvae were used per treatment and all treatments were repeated 3–5 times. Additionally, concentrations of 2.5, 1.0, 0.5, 0.25, and 0.1 μg/g diet were included to establish the LC_50_ values. An artificial diet with Cry3Aa was used as positive control at the same concentrations. Additionally, artificial diets containing solubilization buffer, Cry1Ab (50 μg/g diet) or proteins from *E. coli* BL21 cell extract (50 μg/g diet) were used as negative controls. The insect mortality was scored at 5, 10, and 15 days. The POLO-PC software (LeOra Software, 1987) was used for Probit analysis to estimate 50% lethal concentrations The LC_50_ values were considered significantly different if their 95% fiducial limits (FL95) did not overlap.

### Brush Border Membrane Vesicles and Midgut Juice Preparation

Brush border membrane vesicles from *C. puncticollis* whole last-instar larvae were prepared by the differential magnesium precipitation method (Wolfersberger et al., 1987) as modified by Escriche et al. (1995). Protein concentration in the BBMV preparation was determined by the method of Bradford (1976) using BSA as standard. Leucine aminopeptidase (L-APN) was used as a membrane enzymatic marker for the BBMV preparations. The L-APN activity was measured as described previously (Hernández et al., 2004). The specific activity of the L-APN in the BBMV preparation was enriched approximately eightfold relative to the crude homogenate [8.8 ± 0.3 and 1.1 ± 0.2 μmol min^–1^ mg^–1^ protein (mean ± standard deviation), respectively].

Midgut juice was obtained from the *C. puncticollis* last-instar larvae. Prior to dissection, *C. puncticollis* larvae were chilled on ice and their peritrophic membranes containing food were collected and transferred into a 1.5-ml ice-cold Eppendorf tube. The collected guts were centrifuged at 16,000 × *g*, for 10 min at 4°C. The supernatant was immediately distributed in small aliquots, frozen in liquid nitrogen, and kept at −80°C till use. Prior to use, the total amount of proteins in the *C. puncticollis* midgut juice was quantified by Bradford (1976).

### Labeling of Cry Proteins

Trypsin-activated Cry proteins were biotinylated using a protein biotinylation kit (GE HealthCare) according to the manufacturer’s instructions. Prior to labeling, proteins were dialyzed overnight at 4°C in 40 mM Na_2_CO_3_-NaHCO_3_ buffer (pH 8.6). After biotin labeling, the mixture was loaded onto a PD10 desalting column (GE HealthCare) equilibrated with PBS. The eluted fractions were analyzed by 12% SDS-PAGE.

### Binding Assays

Prior to use, BBMV were centrifuged for 10 min at 16,000 × *g* and suspended in binding buffer (PBS, 0.1% BSA, pH 7.4). Competition experiments were performed incubating 50 ng of the biotinylated proteins with 20 μg of BBMV in binding buffer. Incubations were carried out for 1 h at 25°C in the absence or presence of an excess of unlabeled proteins (200-fold excess) in a final volume of 100 μl. After incubation, samples were centrifuged at 16,000 × *g* for 10 min and the pellets were washed with 500 μl of ice-cold binding buffer. The final pellets, containing the bound biotinylated proteins, were suspended in 10 μl of the same buffer and analyzed by 12% SDS-PAGE. The separated proteins were electro-blotted onto a nitrocellulose membrane (HybondTM-ECLTM, GE HealthCare). Biotinylated proteins were visualized after probing with streptavidin-conjugated horseradish peroxidase (1:2000 dilution) with chemiluminescence detection procedure (RPN2109, GE HealthCare) using an ImageQuant LAS4000 image analyzer. Each competition experiment repeated a minimum of three times.

## Results

### Insecticidal Activity of Cry23Aa and Cry37Aa Against *C. puncticollis* Larvae

An early study (Ekobu et al., 2010) showed that solubilized Cry23Aa/Cry37Aa proteins were toxic to *C. puncticollis* larvae. In order to be able to individually study the toxic activities of the two constituent proteins which are present in the crystal produced by the *B. thuringiensis* strain EG10327 toward this insect pest, the genes encoding for the Cry23Aa and Cry37Aa proteins were individually produced in the *E. coli* strain BL21 and then tested against second instar larvae of *C. puncticollis*.

**FIGURE 1:**
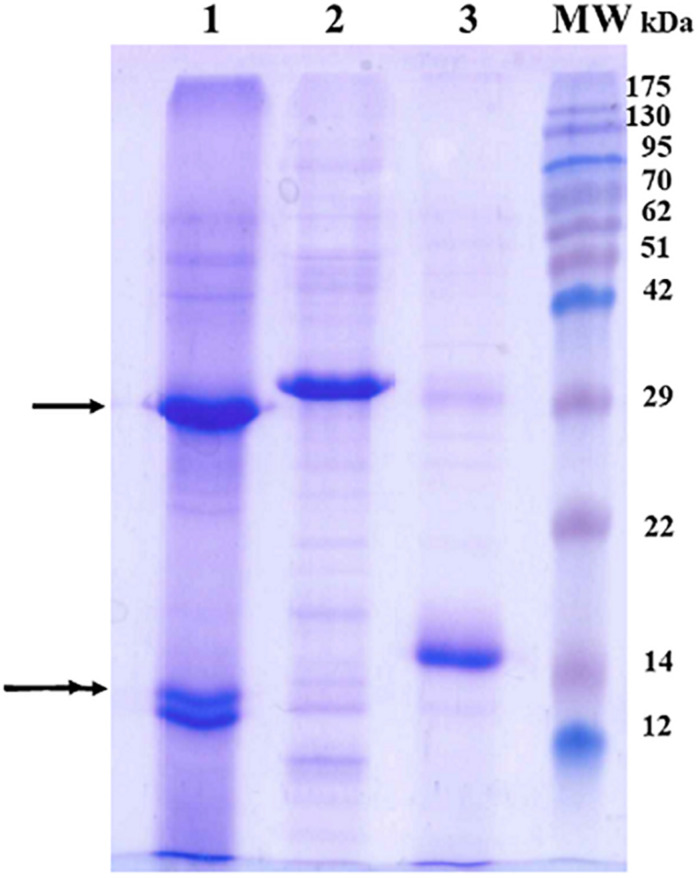
Cry23Aa and Cry37Aa proteins produced by the *B. thuringiensis* strain EG10327 and by recombinant *E. coli* strains. Crystal or inclusion bodies solubilized, Cry23Aa and Cry37Aa proteins from *B. thuringiensis* (lane 1), Cry23Aa from *E. coli* (lane 2), Cry37Aa from *E. coli* (lane 3). MW, molecular mass marker (Pink pre-stained protein ladder, Nippon genetics). The single and double arrowheads indicate the Cry23Aa and Cry37Aa full-length proteins, respectively.

The production of the recombinant Cry23Aa and Cry37Aa proteins was confirmed by both SDS-PAGE and protein fingerprinting (Figure 1 and Supplementary Table S1, respectively) and eventually compared to the Cry23Aa and Cry37Aa proteins expressed in *B. thuringiensis* strain EG10327. The *E. coli*-produced proteins have similar relative molecular masses (approximately 30 and 14 kDa for the Cry23Aa and Cry37Aa, respectively) as the solubilized crystal proteins produced by *B. thuringiensis*. It is worth noting that due to the addition of His-tag tail, the molecular sizes of the Cry23Aa and Cry37Aa proteins produced in the recombinant *E. coli* cells are slightly higher than the proteins produced by *B. thuringiensis* (Figure 1). Moreover, the results from the protein fingerprinting allowed us to putatively identify the SDS-PAGE bands based on the Cry23Aa and Cry37Aa sequences (GenBank Accession No. AAF76375.1 and AAF76376.1, respectively). Moreover, the protein fingerprinting detected the specific peptides for Cry23Aa and Cry37Aa proteins whether expressed in *E. coli* cells or *B. thuringiensis* strain. The bands were identified base on the Cry23Aa and Cry37Aa sequences with sequence coverages of 42 and 73%, respectively (Supplementary Table S1). MASCOT scores gave a high level of confidence that the identifications were correct. The fingerprinting results indicated that the band of *ca.* 30 kDa matched with sequences from the N-terminal region of the protein, starting at the N-terminus and ending at K-261. The fingerprint of the band of approximately 14 kDa consisted of a polypeptide starting at amino acid residue K-28 and ending at K-120.

According to the results of bioassays, Cry37Aa, and to a lesser extent Cry23Aa, are toxic to *C. puncticollis* larvae, when they are produced individually in recombinant *E. coli* cells (Table 1). In addition, solubilized Cry3Aa is also active against *C. puncticollis*, whereas the lepidopteran specific Cry1Ab protein and the proteins present in the bacterial culture extract from BL21, used as negative controls, are inactive against *C. puncticollis* larvae (Table 1).

**TABLE 1:** Toxicity of Cry3Aa, Cry23Aa, Cry37Aa, and Cry23Aa/Cry37Aa proteins against *C. puncticollis* larvae after 15 days.

**Protein**	**LC_50_ (μg/g)^a^**	**Fiducial limits 95% (μg/g)**		**Slope ± SE**
		**Lower**	**Upper**	
Cry23Aa^b^	2.12	1.17	5.75	0.97 ± 0.18
Cry37Aa^b^	1.25	0.77	2.22	1.12 ± 0.18
Cry23Aa/Cry37Aa^c^	1.37	0.78	2.26	1.21 ± 0.22
Cry3Aa^c^	2.72	1.64	6.29	0.96 ± 0.20
Cry1Ab^b^	>50	–	–	–
Proteins from the BL21 cell extract	>50	–	–	–

*^*a*^LC_50_, 50% lethal concentration. ^*b*^Proteins obtained from recombinant *E. coli* strains were produced individually. ^*c*^Cry3Aa and Cry23Aa/Cry37Aa were obtained from the *B. thuringiensis* strains 4AA1 and EG10327, respectively.*

### Proteolytic Processing of Cry23Aa/Cry37Aa Protoxins

**FIGURE 2:**
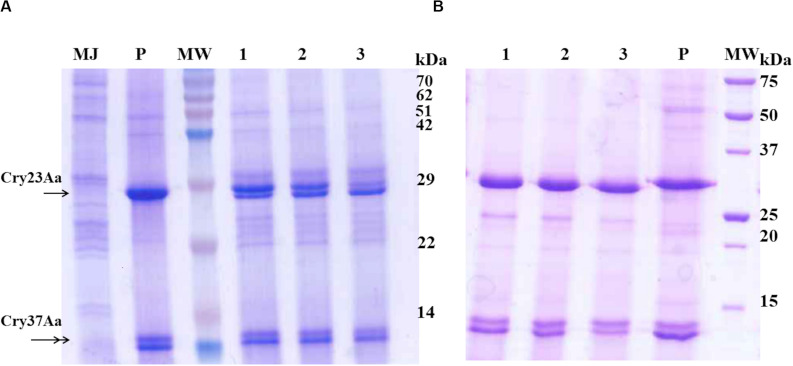
Time course of proteolytic processing of the solubilized Cry23Aa/Cry37Aa protoxins from *B. thuringiensis* by *C. puncticollis* midgut juice **(A)** and trypsin **(B)**. Solubilized Cry23Aa/Cry37Aa proteins were incubated for 30 min (lanes 1), 60 min (lanes 2), and 180 min (lanes 3). MW, molecular mass marker (in kDa). MJ, proteins present in the midgut juice from *C. puncticollis*. P, solubilized Cry23Aa/Cry37Aa (6 μg) proteins without treatment. The single and double arrowheads indicate the Cry23Aa and Cry37Aa proteins, respectively.

In general, Cry proteins are produced as protoxins and are processed by the action of midgut proteases of susceptible insects to render the active form, which has receptor-binding and pore-forming abilities. Here, we analyzed the proteolytic pattern obtained after the treatment of solubilized Cry23Aa/Cry37Aa crystal proteins from *B. thuringiensis* with midgut juice proteases from *C. puncticollis* and the commercial enzyme trypsin. Time course assays showed the ability of the gut fluid proteases to full-activated the Cry23Aa protoxin (approximately 30 kDa) to a protease-resistant core with a molecular weight of *ca*. 29 kDa, whereas minimal or no observable effect on the processing of the Cry37Aa protoxin was observed (Figure 2A). A similar proteolytic pattern was observed when Cry23Aa/Cry37Aa proteins were treated with trypsin (Figure 2B). Altogether, these results point out that proteolytic processing occurs for the Cry23Aa but not for the Cry37Aa.

### Size Exclusion Chromatography Revealed an Interaction Between Cry23Aa and Cry37Aa

**FIGURE 3:**
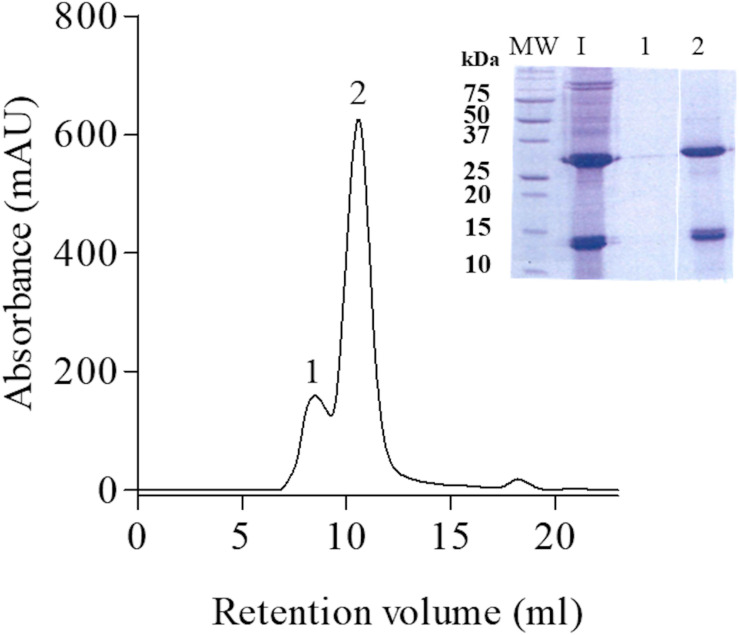
Gel filtration chromatography of the Cry23Aa/Cry37Aa proteins from *B. thuringiensis* after treatment with trypsin. The sample was injected into a Superdex 75 10/300 GL column at a flow rate of 0.5 ml/min. The proteins present in the peaks 1 and 2 are shown in the SDS-PAGE. MW, molecular weight markers (kDa). I, input: sample injected in the column. Absorbance was measured at 280 nm.

To investigate the interaction between trypsin-activated Cry23Aa/Cry37Aa components produced in *B. thuringiensis* strain in solution, gel filtration chromatography was carried out with a Superdex 75 10/300 GL column. The chromatogram revealed a single major high molecular weight peak which corresponds to a protein of size of approximately 44 kDa (Figure 3), which matches the size of both proteins together. This result shows that, under native conditions, Cry23Aa and Cry37Aa are interacting somehow forming a complex as they co-elute in the same peak. The analysis of this peak by SDS-PAGE showed two main bands of about 29 kDa and 14 kDa (Figure 3, lane 2 in inset). Other chromatographic peaks were of high molecular weight, though no protein was detected after analysis by SDS-PAGE (Figure 3, peak 1 corresponding to lane 1).

### Binding Analysis

**FIGURE 4:**
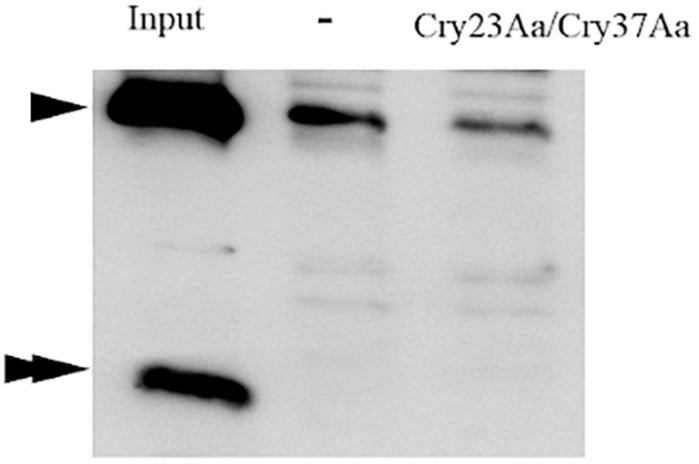
Homologous competition binding assays with BBMV from *C. puncticollis*. Binding of trypsin-activated Cry23Aa/Cry37Aa from *B. thuringiensis* was analyzed in the absence (–) or the presence of 200-fold excess of the unlabeled competitor. Input shows biotinylated Cry23Aa/Cry37Aa proteins. The single and double arrowheads indicate the Cry23Aa and Cry37Aa proteins, respectively.

The mixture of Cry23Aa and Cry37Aa, resulting after trypsin treatment of the solubilized crystals from the *B. thuringiensis* strain EG10327, was labeled with biotin. Homologous competition assays showed that Cry23Aa was able to bind and this interaction was specific since it competed with a 200-fold excess of unlabeled proteins (Figure 4). In contrast, no binding was observed for the Cry37Aa protein.

**FIGURE 5:**
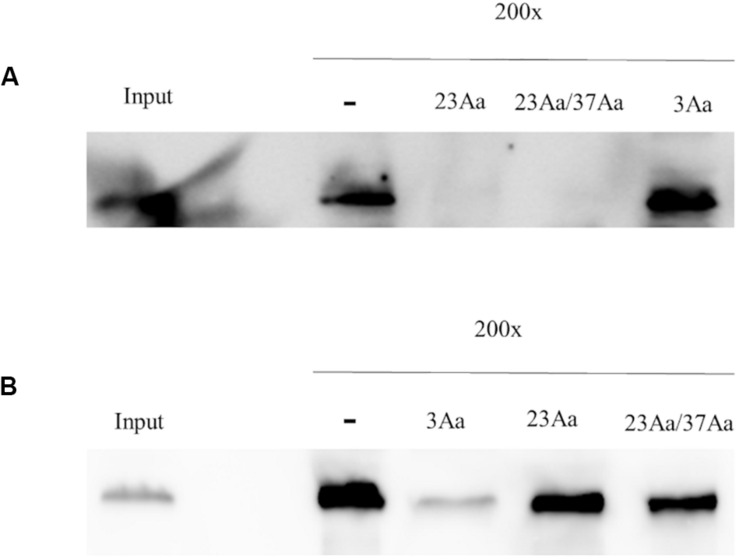
Binding of biotinylated Cry23Aa **(A)**, and Cry3Aa **(B)** proteins to *C. puncticollis* BBMV in the absence of competitor (–) or in the presence of a 200-fold excess of competitors. Lanes labeled as 23Aa/37Aa, 23Aa, and 3Aa correspond to Cry23Aa/Cry37Aa, Cry23Aa, and Cry3Aa proteins, respectively. Input shows biotinylated Cry proteins. The biotinylated proteins used in the experiments were obtained from *B. thuringiensis* strains.

To explore the ability of independently binding of Cry23Aa to *C. puncticollis* BBMV in the absence of Cry37Aa, the two components in Cry23Aa/Cry37Aa mixture were separated by anion-exchange chromatography (Supplementary Figure 1), and Cry23Aa was labeled with biotin and tested for binding. The results showed that trypsin-activated Cry23Aa bound to *C. puncticollis* BBMV, though Cry37Aa was not present in the assay (Figure 5A). Interestingly, the homologous competition of labeled Cry23Aa was observed in the absence or the presence of Cry37Aa. Thus, the results showed that Cry23Aa binds specifically and independently of the presence of Cry37Aa. In addition, Cry3Aa did not reduce the binding of biotinylated Cry23Aa to *C. puncticollis* BBMV (Figure 5A). These data suggest that Cry23Aa does not share binding sites with Cry3Aa.

Specific binding of biotin labeled Cry3Aa to *C. puncticollis* BBMV was observed. Binding of Cry3Aa was substantially diminished when 200-fold excess of unlabeled Cry3Aa was added. However, neither Cry23Aa alone nor the mixture (Cry23Aa/Cry37Aa), could compete for Cry3Aa binding (Figure 5B). These data indicate that Cry3Aa has different binding sites to Cry23Aa alone or as a mixture on *C. puncticollis* BBMV.

## Discussion

Although extensive efforts have been made to control sweet potato weevils, effective control has not been yet achieved. Therefore, innovative strategies are required for the successful control of *C. puncticollis*. Pyramiding different *cry* genes with a different mode of action in Bt sweet potato crops initially proved by binding and toxicity assays, may lead to efficient control of this pest. Some research has been done to identify toxic Cry proteins to *Cylas* spp. larvae (Ekobu et al., 2010). Accordingly, it has been demonstrated that coleopteran-active Cry proteins; Cry3Aa3, Cry3Bb3, Cry3Ca1, Cry7Aa1, and Cry23Aa/Cry37Aa proteins are toxic for different sweet potato weevil species (Morán et al., 1998; Ekobu et al., 2010; Rukarwa et al., 2014). More attention has been directed toward the 3-domain group of insecticidal Cry proteins. In contrast, less is known about the mode of action of non-3-domain toxins such as the Cry23Aa/Cry37Aa proteins belonging to the β-sheet group. Therefore, in the present study, the contribution of each protein in terms of insecticidal activity toward *C. puncticollis* larvae and binding of Cry23Aa/Cry37Aa proteins to BBMV of this insect pest were assessed.

To date, it is generally accepted that Cry23Aa and Cry37Aa proteins act as a binary toxin (Donovan et al., 2001). In fact, it has been claimed that for some coleopteran insect pests such as *Anthonomus grandis*, *Tribolium castaneum*, *Diaprepes abbreviates*, *Popillia japonica*, *C. puncticollis*, *Cylas brunneus*, and *Capnodis* spp. (especially *C. cariosa*), both proteins (Cry23Aa/Cry37Aa) are required for toxicity (Donovan et al., 2001; Weathersbee et al., 2006; Contreras et al., 2013; Gindin et al., 2014). Although none of the previous researches studied the toxicity of Cry23Aa and Cry37Aa separately. In the present study, the Cry23Aa and Cry37Aa proteins were individually produced in *E. coli* cells and tested for their insecticidal activity against second instar larvae of *C. puncticollis*. Moreover, expressing these two genes in *E. coli* allows us to test the activity of both proteins regardless of possible interactions with other compounds that can be present in the native host. The results showed that both recombinant proteins were toxic for *C. puncticollis* larvae when tested individually, suggesting that the presence of both proteins is not necessary to exert their toxicity against *C. puncticollis* larvae.

In the case of other binary toxins, both components are required for the maximal insecticidal activity. The presence of both components of Bin toxin, BinA and BinB, from *Lysinibacillus sphaericus* has been described crucial for toxicity to mosquito larvae, in the sense that BinB was not toxic for *Culex pipiens* when assayed alone (Sebo et al., 1990). However, it has been claimed that BinA alone is sufficient to exert toxicity to *C. pipiens* larvae (Nicolas et al., 1993) and the insect cell culture from *Culex quinquefasciatus* (Baumann and Baumann, 1991). The insecticidal activity of Cry34Ab1 and Cry35Ab1 were assessed individually and in combination against *Diabrotica undecimpunctata howardi*. The obtained results showed that although Cry34Ab1 (a 14-kDa protein) was active against the tested insect and Cry35Ab1 (a 44-kDa protein) caused no mortality, both toxins are required for maximal insecticidal activity (Herman et al., 2002). In the case of Cry48Aa/Cry49Aa proteins, none of the components showed larvicidal activity singly against *Culex* larvae, whilst exhibited a high level of toxicity in combination (Jones et al., 2007). It is noteworthy that the insecticidal activity of the components of different binary toxins can be varied from one insect species to another. In the Cry15Aa/40-kDa complex protein, the 40-kDa protein neither was toxic nor showed synergistic activity for the toxicity of Cry15Aa against the tested lepidopteran insect species, while the second partner, Cry15Aa toxin, has been found toxic (Brown and Whiteley, 1992; Naimov et al., 2008). While, in the case of *Cydia pomonella*, there are contradictory results that claim both the synergistic activity of the 40-kDa protein (Rang et al., 2000) and also mention the lack of contribution of 40-kDa protein in the toxicity of Cry15Aa (Rang et al., 2000; Naimov et al., 2011). However, it has been claimed that the 40-kDa protein is essential for solubilization and crystal formation of Cry15Aa (Naimov et al., 2011). Another exception in the β-sheet toxins group is the Cry36 protein which exerts its toxicity against *Diabrotica* larvae without a partner (Rupar et al., 2000). Therefore, to determine if the insecticidal activity of Cry23Aa and Cry37Aa relies on the insect species, the toxicity of each component has to be evaluated individually against other susceptible hosts and compared to the toxicity of Cry23Aa/Cry37Aa.

Homologous competition assays showed that the binding of Cry23Aa protein to *C. puncticollis* BBMV is specific. Moreover, Cry37Aa is not required for the binding of Cry23Aa since it can bind specifically to *C. puncticollis* BBMV without Cry37Aa. Interestingly, no binding was detected for the Cry37Aa protein, when the protein mixture (Cry23Aa/Cry37Aa) was labeled with biotin (Figure 4). The binding behavior of the Cry37Aa protein to *C. puncticollis* BBMV has not been addressed in the present study due to the interference of biotin labeling in the biological activity of this protein (unpublished data). In agreement, it was previously reported that the iodine labeling of Cry34Ab1 abolished both the insecticidal activity of this protein and the specific binding to western corn rootworm BBMV (Li et al., 2013). Therefore, further experiments are needed to thoroughly characterize the mode of action of Cry37Aa protein.

Heterologous binding assays using biotinylated proteins demonstrated that Cry23Aa alone or in the combination with Cry37Aa does not compete with Cry3Aa for the same binding sites on *C. puncticollis* BBMV. Interestingly, previous studies have shown common binding sites for Cry3Ba, Cry3Ca, and Cry7Aa on *C. puncticollis* BBMV (Hernández-Martínez et al., 2014). Based on binding site interaction results of Cry3Aa and Cry3Ca proteins probably have two different binding sites that one of them is common between these two proteins (Hernández-Martínez et al., 2016). Thus, these observations along with the structural difference and subsequently different mechanisms of action of Cry23Aa to other conventional three-domain Cry proteins support Cry23Aa as a desirable candidate to be pyramided with Cry3Aa in Bt sweet potatoes to control of *C. puncticollis* effectively and delay the evolution of resistance.

Our study provides the evidence for toxicity of Cry23Aa and Cry37Aa proteins individually against *C. puncticollis* larvae. Moreover, according to our results, Cry23Aa binds to *C. puncticollis* BBMV specifically and independently of Cry37Aa. Due to the lack of common binding sites, Cry23Aa protein can be pyramided with Cry3Aa protein for better management of *C. puncticollis*. Therefore, expressing non-homologous Bt Cry proteins in sweet potato would be a great strategy of controlling *C. puncticollis*.

## Data Availability Statement

The raw data supporting the conclusions of this article will be made available by the authors, without undue reservation.

## Author Contributions

PH-M, AK, KP, AA-G, and NV-V performed the experiments. PH-M, AK, and BE analyzed the data. PH-M and AK wrote the manuscript. PH-M and BE conception, designed, and supervised the work. All authors reviewed and edited the manuscript.

## Conflict of Interest

The authors declare that the research was conducted in the absence of any commercial or financial relationships that could be construed as a potential conflict of interest.
