# Supplementary figures and images for: The Independent Biological Activity of Bacillus thuringiensis Cry23Aa Protein Against Cylas puncticollis
Source: Front Microbiol. 2020 Jul 22;11:1734. doi: 10.3389/fmicb.2020.01734 (PMC7387505; doi:10.3389/fmicb.2020.01734)

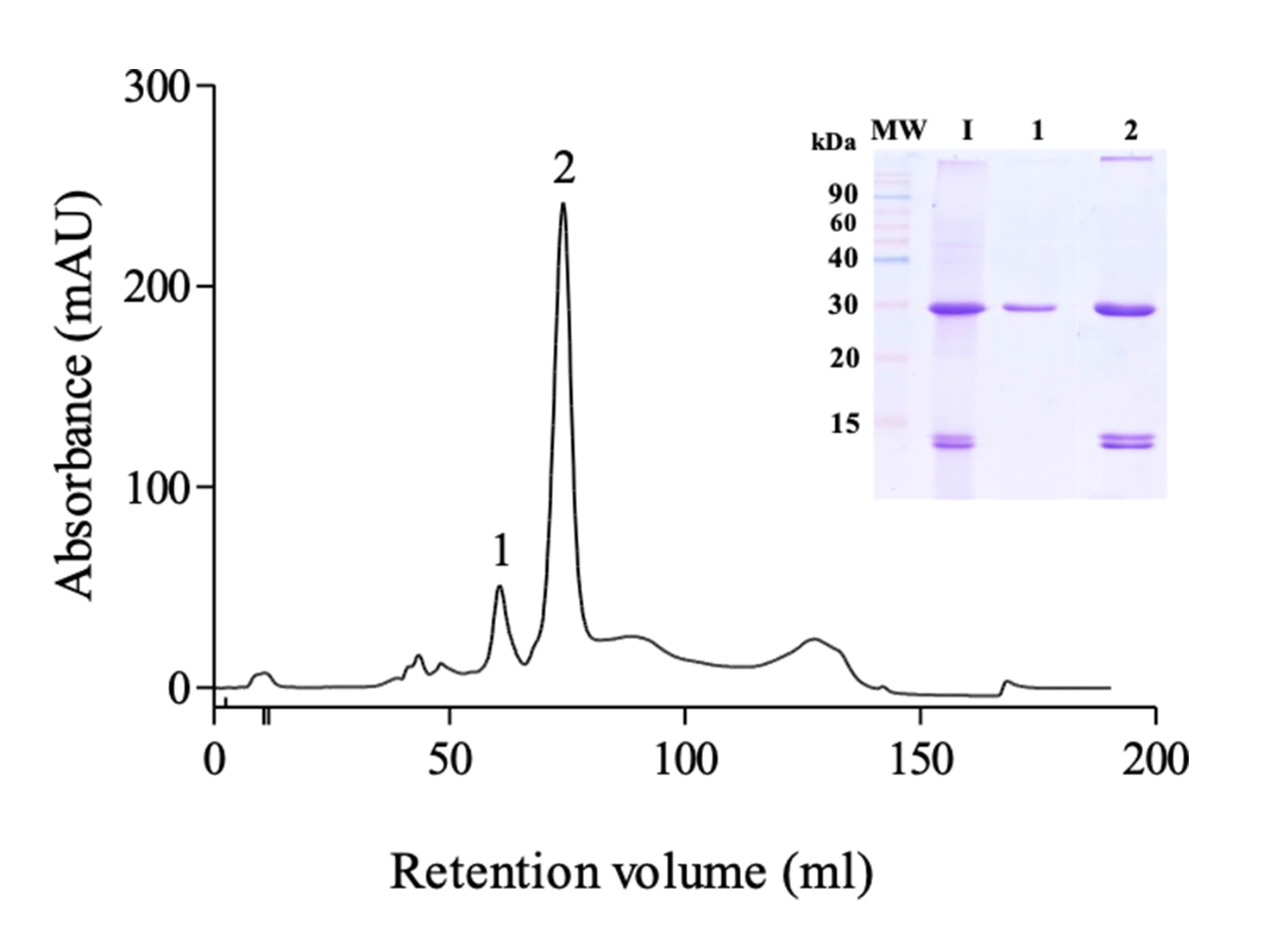

Supplement: Supplementary file 1 [file Image_1.tif]
