# Supplementary material for: The Independent Biological Activity of Bacillus thuringiensis Cry23Aa Protein Against Cylas puncticollis
Source: Front Microbiol. 2020 Jul 22;11:1734. doi: 10.3389/fmicb.2020.01734 (PMC7387505; doi:10.3389/fmicb.2020.01734)
Supplement: Supplementary file 2 [file Table_1.docx]

**Supplementary table**

**Table S1.** Template data from protein mass fingerprinting found by MASCOT search

| **Protein** | **Genbank accession** | **pI** | **M_r_** | **Coverage** | **MASCOT score** | **Match to** | **Length** |
| --- | --- | --- | --- | --- | --- | --- | --- |
| Bt Cry23Aa | AAF76375.1 | 4.92 | 29.309 | 44% | 301 | gi\|8567977 | 267 |
| Bt Cry37Aa | AAF76376.1 | 4.42 | 14.635 | 73% | 148 | gi\|557884471 | 126 |
| *E. coli* Cry23Aa | MK248953 | 4.92 | 29.309 | 42% | 228 | gi\|8567977 | 267 |
| *E. coli* Cry37Aa | MK248954 | 4.42 | 14.258 | 73% | 266 | gi\|557884471 | 126 |
